# Reply to the Comment from Watson, K.M. “Letter to the Editor Re: Kipperman, B.S. and German, A.J. *Animals* 2018, *8*, 143” *Animals* 2018, *8*, 179

**DOI:** 10.3390/ani8110202

**Published:** 2018-11-09

**Authors:** Barry S. Kipperman, Alexander J. German

**Affiliations:** 1 Department of Veterinary Medicine and Surgery, University of Missouri College of Veterinary Medicine, Columbia, MO 65211, USA; 2 Institute of Ageing and Chronic Disease and Institute of Veterinary Science, University of Liverpool, Neston CH64 7TE, UK; ajgerman@liverpool.ac.uk

**Keywords:** companion animal, obesity, overweight, One Health

Dear Editor,

We appreciate the interest [1] in our opinion paper [2] regarding companion animal obesity and, although there is limited published evidence, agree that some pet owners might respond negatively when this issue is addressed. As a One Health problem, we are not surprised that scientific literature corroborates under-reporting of obesity by human health care providers and common barriers to discussing this disease. We agree that further studies are warranted to encourage and assist veterinarians in both reporting and discussing overweight and obesity.
